# Characterization of the Nqo5 subunit of bacterial complex I in the isolated state

**DOI:** 10.1002/2211-5463.12070

**Published:** 2016-06-08

**Authors:** Yuya Hanazono, Kazuki Takeda, Kunio Miki

**Affiliations:** ^1^ Department of ChemistryGraduate School of ScienceKyoto UniversitySakyo‐kuKyotoJapan; ^2^ RIKEN SPring‐8 Center at Harima InstituteSayoHyogoJapan

**Keywords:** 30‐Kd subunit motif, crystal structure, NADH:ubiquinone oxidoreductase

## Abstract

The subunits that comprise bacterial complex I (NADH:ubiquinone oxidoreductase) are also found in more complicated mitochondrial enzymes in eukaryotic organisms. Although the Nqo5 subunit is one of these conserved components and important for the formation of complex, it has been little studied. Here, we report structure analyses of isolated Nqo5 from *Thermus thermophilus*. Biochemical studies indicated that the C‐terminal region following the 30‐Kd subunit motif is disordered in the isolated state, while the remaining portion is already folded. Crystallographic studies of a trypsin‐resistant fragment revealed detailed structural differences in the folded domain between the isolated and complexed states.

**Abbreviations**
CD: circular dichroism
FOM: figure of merrit
MAD: multi‐wavelength anomalous diffraction
NADH: reduced form of nicotinamide adenine dinucleotide
PAGE: polyacrylamide gel electrophoresis

NADH:ubiquinone oxidoreductase (complex I) is one of the respiratory chain components (complexes I–IV and ATP synthase) in the mitochondrial inner membrane of eukaryotes or the plasma membrane of prokaryotes. The enzyme carries out an electron transfer reaction from NADH to ubiquinone, and makes a proton concentration gradient across the membrane by proton pumping. More than 40 subunits compose the complex I of mammalian mitochondria, and the resultant molecular mass is about 1000 kDa. On the other hand, bacterial complex I (~ 500 kDa) has a simpler composition with 13–15 subunits. Respiratory complexes from the thermophilic bacterium *Thermus thermophiles* have frequently been used in the structural studies because of their high stability 1, 2, 3, 4. The whole structure of bacterial complex I, including the transmembrane subunits was first determined at 3.3 Å resolution 5, while eukaryotic complexes I from *Yarrowia lipolytica* and *Bos taurus* were reported more recently 6, 7.

A subunit with a molecular weight of 30 kDa (30 Kd subunit for the bovine enzyme, NDUFS3 for the human enzyme) is encoded in nuclear DNA in the case of most eukaryotes 8. The subunit has a signature pattern of EREx_2_(D/E)(L/I/V/M/F/Y)_2_x_6_(H/K)x_3_(K/R/P)x(L/I/V/M)(L/I/V/M/Y/S) in the C‐terminal region, which is known as the respiratory‐chain NADH dehydrogenase 30‐Kd subunit signature. The subunit is contained in all homologous enzymes, and the human homolog (NDUFS3) is involved in diseases 9, 10, 11. The bacterial homologous proteins are denoted as Nqo5 (for *T. thermophilus* and *Paracoccus denitrificans*) or NuoC (for *Escherichia coli*). The subunit composes the hydrogenase‐like module together with the Nqo4, Nqo6, and Nqo9 subunits. Homologous proteins of the Nqo5 subunit are also contained in membrane‐bound multisubunit hydrogenases 12, while soluble‐type hydrogenases are not. It has been reported that the subunit has a central role in the assembly of complex I 13, 14, 15, 16, 17, 18. Although the structure of the Nqo5 subunit in the complexed state has been elucidated, the details of the assembly process remain unclear.

In this paper, we report structural studies of the conserved subunit Nqo5 of the bacterial complex I from *T. thermophilus*. Strictly folded and flexibly extended portions of the Nqo5 subunit in the isolated state are distinguished according to biochemical and crystallographic results. The implications for roles in assembly process and conformational changes of the whole complex are discussed based on a comparison of Nqo5 structures in the isolated and complexed states.

## Materials and methods

### Expression and purification

The *nqo5* gene from the genome of *T. thermophilus* HB8 was ligated into pET11a (Novagen, Madison, WI, USA). The transformed *Escherichia coli* strain BL21 (DE3) was grown at 310 K in LB medium and 0.1 mm of ampicillin. Cultured cells were broken by sonication, and the cell membrane was removed by centrifugation (10 000 ***g***). The supernatant was heated at 343 K. The centrifuged supernatant was successively applied to a Super Q Toyopearl 650M column (Tosoh, Tokyo, Japan), Resource Q column (GE Healthcare, Buckinghamshire, UK), CHT5‐I hydroxyapatite column (Bio Rad, Hercules, CA, USA), Superdex200 (GE Healthcare) column, and HiPrep desalting column (GE Healthcare). Selenomethionyl Nqo5 was expressed in B834(DE3), and purified in a manner similar to the native protein.

### Protease degradation assays

Purified Nqo5 was digested with trypsin in a buffer solution (50 mm Tris‐HCl, pH 8.0). The ratio of the weight of protein to protease was 100 : 1. The solutions were incubated at 277 K, and were sampled at 0, 1, 2, 4, and 24 h. The reaction was terminated by adding PAGE buffer and immediately stored at 253 K. SDS/PAGE analyses were performed with 15% gels. Gels were stained with Coomassie brilliant blue. The trypsin‐resistant fragment with a molecular mass of 15.6 kDa was purified with a size exclusion column, Superdex75 (GE Healthcare).

### Mass spectrometry

The masses of full‐length Nqo5 and the fragment of Nqo5 digested by trypsin at 277 K for 24 h were determined by matrix‐assisted laser desorption ionization mass spectrometry (MALDI‐MS) (Voyager‐DE RP; Applied Biosystems, Waltham, MA, USA). Digested protein solution was desalted with ZipTip C18 (Merck Millipore, Billerica, MA, USA) for MALDI‐MS experiments. 10 μL of the desalted solution was mixed with 4.0 μL of a solution containing 10 mg·mL^−1^ sinapinic acid, 0.1% trifluoroacetic acid, and 50% acetonitrile.

### Circular dichroism spectroscopy

Circular dichroism (CD) spectra were measured using a J‐805 spectropolarimeter (Jasco, Tokyo, Japan). The samples of the full‐length and trypsin‐resistant fragment of Nqo5 (0.1 mg·mL^−1^) were dissolved in 5.0 mm of potassium phosphate buffer in a 1.0‐mm quartz cuvette. All CD spectra were measured at 293 K. Five scans were averaged for each spectrum. The secondary structure contents were determined with the JWSSE‐408 program (Jasco) using a reference data set 19.

### Crystallization

The 15.6‐kDa fragment as well as the full‐length protein was used for crystallization experiments with the sitting drop vapor diffusion method at 293 K. High‐quality crystals were obtained only from the fragment. Rod‐shaped orthorhombic crystals with typical dimensions of 0.3 × 0.1 × 0.1 mm^3^ were grown in crystallization solutions which consisted of a protein solution (5 mg·mL^−1^ protein, 20 mm Tris–HCl pH 8.0 and 5% (w/v) NDSB‐195) and a reservoir solution (200 mm calcium chloride and 25% (w/v) PEG4000). Hexagonal crystals with typical dimensions of 0.1 × 0.1 × 0.1 mm^3^ were grown from a crystallization solution that consisted of the protein solution and a reservoir solution (10% (w/v) PEG1000 and 10% (w/v) PEG8000). Selenomethionine‐derivative crystals were obtained under the same conditions as the Native I crystal.

### X‐ray data collection

Native data sets (Native I and II) of the wild‐type protein and three wavelength data sets of the selenomethionyl protein were collected at 95 K. Diffraction data sets were collected at SPring‐8, and processed and scaled using the HKL2000 package 20. The space group of the Native I crystal is *P*2_1_2_1_2_1_ with unit cell parameters of *a* = 41.3 Å, *b* = 42.2 Å, *c* = 70.1 Å. The space group of the Native II crystal is *P*6_3_ with unit cell parameters of *a* = *b* = 55.2 Å, *c* = 71.8 Å. The selenomethionyl derivative crystal belongs to the space group *P*2_1_2_1_2_1_ and the unit cell parameters are *a* = 41.0 Å, *b* = 42.1 Å, *c* = 69.9 Å. The crystallographic statistics are listed in Table 1.

**Table 1:** Data collection and phasing statistics

	Se‐Met			Native I	Native II
	Lamda‐1	Lamda‐2	Lamda‐3		
Data collection	Peak	Inflection	Remote		
Wavelength (Å)	0.9796	0.9801	0.9900	1.0000	1.0000
Temperature (K)	95			95	95
Crystal data					
Space group	*P*2_1_2_1_2_1_			*P*2_1_2_1_2_1_	*P*6_3_
Cell parameters					
*a* (Å)	41.0			41.3	55.2
*b* (Å)	42.1			42.2	55.2
*c* (Å)	69.9			70.1	71.8
Resolution range (Å)	40–1.70 (1.76–1.70)	40–1.70 (1.76–1.70)	40–1.70 (1.76–1.70)	50–1.65 (1.71–1.65)	40–3.00 (3.10–3.00)
Reflections (total/unique)	78 306/13 255	87 294/13 314	86 533/16 297	184 636/14 331	19 936/2499
Completeness (%)	95.5 (77.2)	95.8 (76.5)	95.5 (77.6)	93.8 (70.6)	98.4 (89.3)
*I/*σ (*I*)	22.3 (2.4)	22.7 (2.2)	21.8 (1.8)	33.1 (1.8)	10.7 (1.4)
*R*_sym_ a (%)	6.0 (22.0)	6.1 (37.4)	5.2 (32.2)	5.2 (34.8)	9.6 (29.8)
Phasing					
FOM by SOLVE	0.35				
FOM after RESOLVE	0.55				

Values in parentheses refer to the highest resolution shell.

a *R*_sym_ = Σ_hkl_Σ_i_|*I*_hkl,i_‐<*I*_hkl_>|/Σ_hkl_Σi*I*_hkl,i._

### Structure determination and refinement

**Figure 1:**
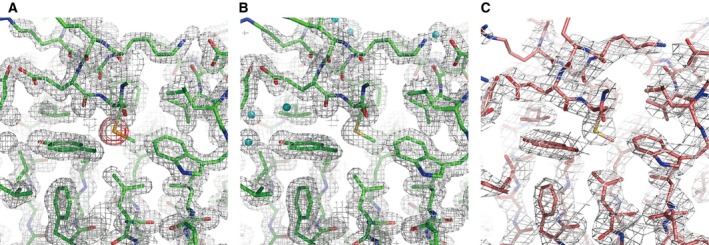
Electron density maps. (A) Experimental electron density map after density modification for selenomethionine‐derivative Nqo5 at 1.7 Å resolution is represented as gray mesh at a contour level of 1.2σ. The anomalous difference map indicating positions of selenium atoms is shown at the 5σ level as red mesh. The refined model of the derivative is also shown as a stick model. (B) The sigma‐A weighted 2*F*_obs_‐*F*_calc_ map for the Native I crystal at 1.65 Å resolution is represented as gray mesh at a contour level of 1.2σ. (C) The sigma‐A weighted 2*F*_obs_‐*F*_calc_ map for the Native II crystal at 3.0 Å resolution is represented as gray mesh at a contour level of 1.2σ.

The phasing was performed by the multi‐wavelength anomalous diffraction (MAD) method with the phenix.autosol 21. The figure of merit (FOM) was 0.35 for 40–1.7 Å. The FOM was improved to 0.55 after the density modification. The electron density map was adequate for the model building (Fig. 1A). Autotracing was carried out with the phenix.autobuild 22. The output structure was subjected to further manual improvement with the Coot program 23. The structure was refined against the Native I data set using the phenix.refine 22 (Fig. 1B). The final *R*_work_ and *R*_free_ factors were 16.9% and 20.9%, respectively. The structure for the Native II crystal was determined by the molecular replacement method with the MOLREP program 24 in the CCP4 suite 25 using the Native I structure as a search model. The *R*‐factor and the correlation coefficient are 38% and 0.61, respectively. The structure was refined with the phenix.refine (Fig. 1C). The final *R*_work_ and *R*_free_ factors were 19.5% and 23.4%, respectively. The structures were validated with the Molprobity program 26. The refinement statistics for Native I and II are listed in Table 2. Figures were prepared using the PyMOL program 27. The coordinates and structural factors for the Native I and II crystals have been deposited in the Protein Data Bank under accession numbers PDB: 5B3P and PDB: 5B3Q, respectively.

**Table 2:** Refinement statistics

Data set	Native I	Native II
Space group	*P*2_1_2_1_2_1_	*P*6_3_
Resolution (Å)	40–1.65	40–3.0
Modeled residues	134	132
Water molecules	120	0
Other heterogen atoms	3 × Ca^2+^	0
Total atoms	1256	1087
*R*_work_ a/*R*_free_ b (%)	17.0/20.9	19.5/23.4
Average *B*‐factor (Å^2^)	26.5	36.7
Rmsd		
Bond (Å)	0.013	0.003
Angle (°)	1.57	0.76
Ramachandran plot (%)		
Favored	97.1	96.9
Allowed	2.9	3.1
Outliers	0	0
PDB code	5B3P	5B3Q

a *R*_work_ = Σ_hkl_||*F*_obs_|‐|*F*_calc_||/Σ_hkl_|*F*_obs_|.

b *R*_free_ was calculated using 5% of the reflections that were not included in the refinement as a test set.

## Results

### Characterization of the Nqo5 subunit in the isolated state

**Figure 2:**
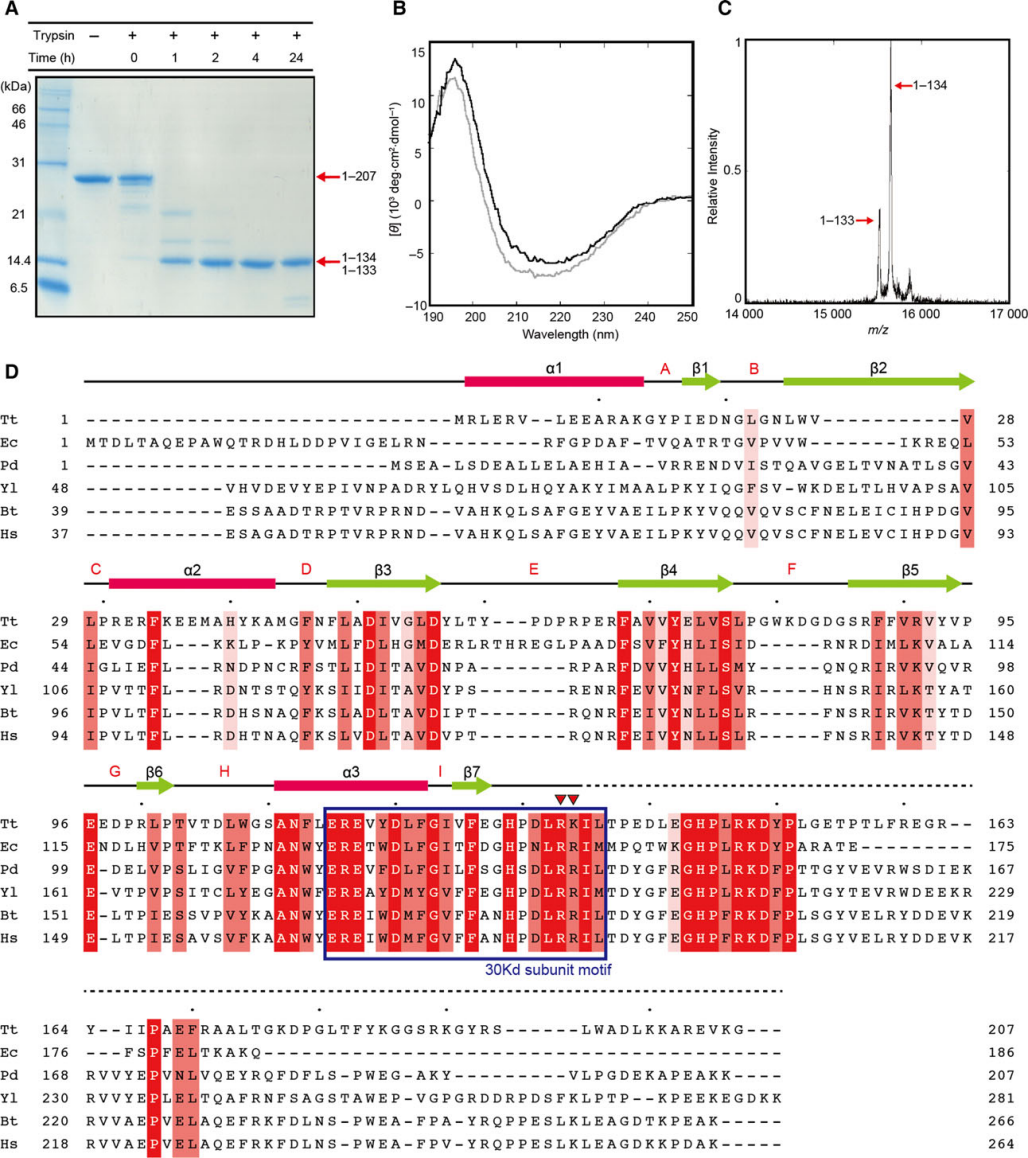
Properties of Nqo5 in the isolated state. (A) The SDS/PAGE analysis of the Nqo5 subunit digested by trypsin. Masses of fragments measured by MALDI‐MS are indicated together with assignments. (B) CD spectra of the Nqo5 subunit. The gray and black lines indicate spectra of the full‐length protein and the protease‐resistant fragment of 15.6 kDa (residue 1–134). (C) Mass spectrum of Nqo5. The black line indicates the spectrum of the fragments of Nqo5 digested by trypsin. (D) Sequence alignment. Completely conserved residues, highly conserved residues, and relatively conserved residues are filled with red, light red, and pale red, respectively. The α‐helices are indicated by cylinders and β‐strands by arrows. Digestion sites by trypsin are indicated by red triangles. Tt, *Thermus thermophilus*; Pd, *Paracoccus denitrificans*; Ec, *Escherichia coli*; Yl, *Yarrowia lipolytica*; Bt, *Bos taurus* (bovine); Hs, *Homo sapiens* (human).

The Nqo5 subunit from *T. thermophilus* was expressed in *E. coli*, and obtained as a soluble protein. In order to elucidate the conformation in the isolated state, protease accessibility assays and circular dichroism (CD) spectroscopy were performed. A fragment with a molecular mass of 15.6 kDa was remained even after long incubation with trypsin (Fig. 2A). The trypsin‐resistant fragment was purified for further characterization. The CD spectrum for the fragment is different from that for the whole molecule (Fig. 2B). The content for random conformation is decreased from 17% to 4% by the truncation. The digestion sites of the fragment were determined to be Arg133 and Lys134 at the C‐terminal region according to the mass spectrum (Fig. 2C), while the several potential digestion sites exist throughout the molecule (Fig. 2D).

### Structural features of the protease‐resistant fragment

**Figure 3:**
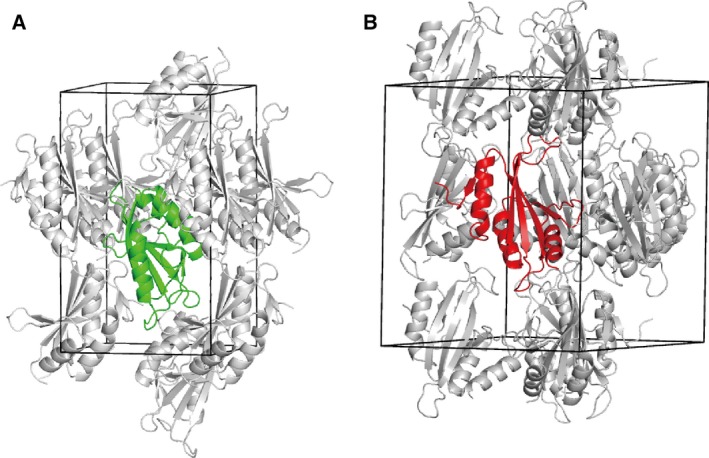
Crystal packing of the Nqo5 subunit. (A) The Nqo5 subunit in the Native I crystal (*P*2_1_2_1_2_1_) is represented as a green ribbon, while symmetry‐related molecules are in gray. The unit cell boundaries are defined as black lines. (B) The Nqo5 subunit in the Native II crystal (*P*6_3_) is represented as a red ribbon.

**Figure 4:**
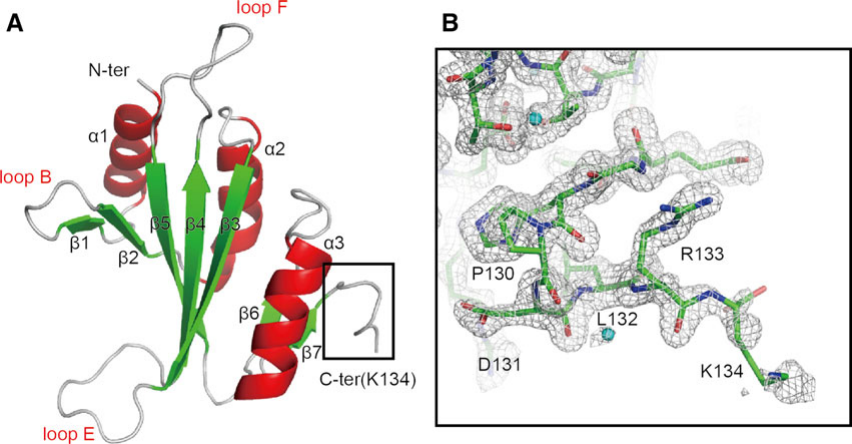
Crystal structure of the protease‐resistant fragment. (A) The crystal structure is shown as a ribbon model, in which α‐helices (α1‐α3) and β‐strands (β1–β7) are shown in red and green, respectively. Loops are denoted as A‐I. (B) A composite omit map of the residues in the 30 Kd subunit signature motif is shown at the 1.0σ level as a gray mesh. The Nqo5 structure is shown as stick model. Water molecules are shown in cyan spheres.

The full‐length protein and the trypsin‐resistant fragment (residue 1–134) were subjected to crystallization trials. High‐quality crystals were obtained from the fragment. The structure was solved by the multiple wavelength anomalous dispersion (MAD) method using the selenomethionyl protein (Table 1), and refined against the native data sets at 1.65 (Native I in *P*2_1_2_1_2_1_) and 3.0 Å (Native II in *P*6_3_) resolutions (Table 2). The two strucutures can be superimposed with a root mean square deviation of 1.7 Å for all atoms. This finding indicated that the atoms are almost identical despite their quite different crystal packing (Fig. 3). We modeled almost all the trypsin‐resistant fragment into the crystal structure (Fig. 4A). The electron density for the last visible residue, Lys134, is weak (Fig. 4B), indicating the residue has a low occupancy. These facts are consistent with the results from the mass spectra of the digested protein, in which the C‐terminal sides of both Arg133 and Lys134 are considered probable digestion sites for trypsin.

**Figure 5:**
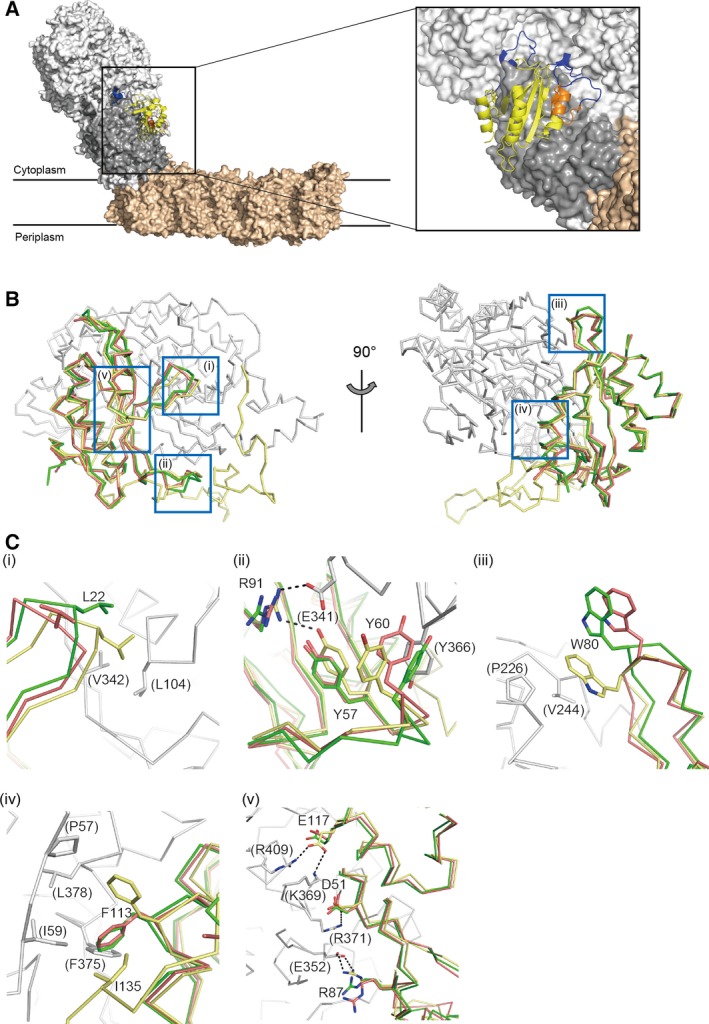
Structural comparison with the subunit in the complex state. (A) The position of the Nqo5 subunit in the complex I structure (PDB: 4HEA). The N‐terminal region of Nqo5 is represented as a ribbon model in yellow. The 30 Kd subunit signature motif is colored in orange. The C‐terminal region of Nqo5 after the 30 Kd subunit motif is represented as blue. Other extramembrane and membrane subunits are represented as white and beige surfaces, while the surface of the Nqo4 is colored in gray. (B) The comparison between the isolated and complexed states. Structures of Nqo5 in the isolated states are colored in green (Native I) and red (Native II), respectively. The structure of Nqo5 in the complex is colored in yellow. The Nqo4 subunit in the complex state is colored in gray. (C) Closed up views around (i) the loop B, (ii) the loop E, (iii) the loop F, (iv) the helix α3, and (v) the interface facing with Nqo4. The labels of residues in parenthesis are for the Nqo4 subunit. Dotted lines in black indicate hydrogen bonds or salt bridges between Nqo4 and Nqo5 in the complex structure.

The structure of the isolated Nqo5 appears to be similar to the subunit in the complexed state (Fig. 5A,B). The rmsd value between the complexed and isolated states is 2.3 Å for all atoms. The loops B, E, and F largely protrude from the main body (Fig. 4A). These regions exhibit significant deviations in the comparison between the isolated and complexed states (Fig. 5B). The loop B has a hydrophobic residue Leu22 at the tip. Leu22 interacts with Leu104 and Val342 of Nqo4 in the complex (Fig. 5C‐i). The loop E has three proline residues (Pro61, Pro63, and Pro65) and two tyrosine residues (Tyr57 and Tyr60) (Fig. 5C‐ii). Tyr60 interacts with both Tyr57 of Nqo5 and Tyr366 of Nqo4 by the aromatic–aromatic interaction in the T‐shaped geometry in the complexed state. Tyr57 as well as Arg91 interact with Glu341 of Nqo4 in the complexed state. Tyr57 and Tyr60 do not interact with each other in the isolated state, while the conformation of Tyr57 is almost identical in both states. Therefore, the conformation of Tyr60 is altered upon the complex formation. The loop F contains hydrophobic residues such as Pro78 and Trp80. The side chain of Trp80 undergoes a hydrophobic interaction with Val244 of Nqo4 in the complexed state (Fig. 5C‐iii). Changes of the side chain conformation are observed for other hydrophobic residues such as Phe113 (Fig. 5C‐iv) at the interface with Nqo4. On the other hand, the conformations of hydrophilic residues such as Asp51, Arg87, Arg91, and Glu117 (Fig. 5C‐ii, v) are similar in both states.

The Nqo5 subunit from *T. thermophilus* has a sequence of ^115^EREVYDLFGIVFEGHPDLRKIL as the respiratory chain NADH dehydrogenase 30 Kd subunit signature. The structure of the signature motif other than Ile135 and Leu136 could be determined in this study. The conformation of this portion is almost identical between the isolated and complexed states. Conserved residues in the signature are at the surface of the subunit with the exception of Glu115. The side chain of Glu115 forms hydrogen bonds with the main chain N atom of Val104 and the side chain of Thr103. Consequently, the protruding portion of the signature is attached to the main body of the subunit. It has been reported that negatively charged residues (Glu115, Glu117, and Asp120) located at the signature sequence are important for the stability of the complex 28.

## Discussion

The present structure indicates that the C‐terminal portion is unfolded or loosely folded in the isolated state, while the N‐terminal portion is already folded. The C‐terminal portion is fixed by interactions with other subunits such as Nqo3, Nqo4, Nqo6, Nqo9, and Nqo16 in the complexed state 5. NuoC (Nqo5) of *E. coli* is fused with NuoD (Nqo4), while these are separately expressed in other species 13, and a large amount of a multimer of NuoCD was observed in the cell lysate of *E. coli* 29. Therefore, it is plausible that Nqo5 initially forms a subcomplex with Nqo4 and accumulates in cells.

Some hydrophobic residues are located at the loop regions. These residues may be involved in the first stage of the complex formation, because these conformations can adapt with high flexibility. The hydrophobic residues are the apparatus for capturing the neighboring Nqo4 subunit. Further associations on the main body surface may be promoted by hydrophobic interactions.

**Figure 6:**
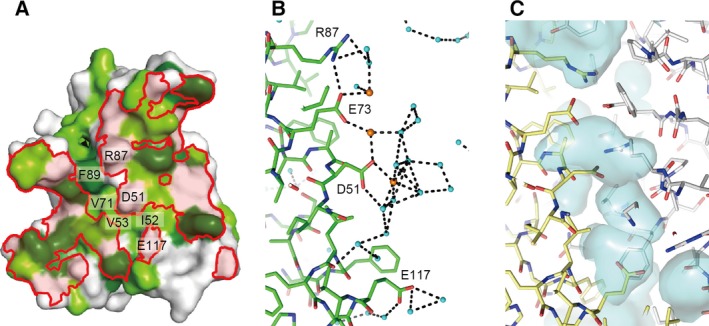
The surface of Nqo5 facing with Nqo4. (A) The hydrophobic property of the molecular surface. The contact areas on the surfaces, which were within 4.0 Å of Nqo4, are enclosed by red boundaries. The orientation is the same as Fig. 4A. The molecular surface (prove radius of 1.4 Å) for aromatic residues (Phe, Trp, and Tyr) are colored in green, and that for other hydrophobic residues (Ala, Ile, Leu, Met, Pro, and Val) in light green. (B) The Nqo5 structure in the Native I crystal is shown as a stick model. Water molecules and calcium ions are shown in cyan and orange spheres. Hydrogen bonds and salt bridges are indicated as black dashed lines. (C) The corresponding portion in the complex is shown as stick model. The Nqo4 and Nqo5 subunits are colored in gray and yellow, respectively. Cavities (prove radius of 1.4 Å) are represented as semitransparent surfaces in cyan.

The interface between Nqo4 also consists of many hydrophilic residues in addition to hydrophobic residues (Fig. 6A). In the complex, hydrophilic residues, such as Asp51, Arg87, and Glu117, interact with Glu352, Lys369, Arg371, and Arg409 of Nqo4 (Fig. 5C‐v). On the other hand, hydrophobic residues, such as Ile52, Val53, Val71, and Phe89, undergo no interaction (Fig. 6A). In the isolated Nqo5, water molecules and calcium ions are located at the positions near the interacting residues of Nqo4 (Fig. 6B). These water molecules bound to the interfacial hydrophilic residues should be removed upon the interaction with the residues of Nqo4. Some water molecules may be left at the interface of the complex, but no such molecules are observed in the complex structure due to the low resolution. Indeed, several cavities are found at the interface between Nqo5 and Nqo4 (Fig. 6C). A high content of hydrophilic residues is unusual at the subunit interface of steady protein complexes. However, some examples can be found in the structure of complexes. The bottom sides of the interfaces of the A and B subunits in the V_1_ complex also have such high contents of hydrophilic residues 4. Each interface of the three AB pairs has a different manner of interaction. This flexibility at the interface is suitable for the open‐close motion in the complex. Similarly, the hydrophilic residues at the Nqo4–Nqo5 interface may be involved in the flexibility of complex I reported in various studies 30, 31, 32, 33, 34. In addition, the possibility of conformational fixation by calcium ions has been pointed out 35. In our experiments, the calcium ions are coordinated with Asp51 and Glu73 in the Native I crystal, which is obtained in the presence of calcium ions (Fig. 6B).

## Author contributions

KM supervised the project. YH and KT performed biochemical and crystallographic analyses. YH, KT, and KM discussed the results and wrote the manuscript.
